# Genetics and epigenetics of alcohol dependence

**DOI:** 10.1186/2049-9256-1-11

**Published:** 2013-07-11

**Authors:** Vanessa Nieratschker, Anil Batra, Andreas J Fallgatter

**Affiliations:** Department of Psychiatry and Psychotherapy, University of Tuebingen, Germany, Calwerstrasse 14, Tuebingen, 72076 Germany

**Keywords:** Genetics, Gene * environment interaction, Epigenetics, DNA methylation, Alcohol dependence, Homocysteine, Withdrawal, Candidate gene studies, Genome-wide studies

## Abstract

Alcohol dependence is a severe and common disorder associated with high morbidity and mortality rates. Genetic as well as environmental factors are known to modulate susceptibility to alcohol dependence. There is a growing body of evidence suggesting that this interaction between the genome and the environment is mediated by epigenetic mechanisms, e.g. DNA methylation at CpG sites. Following an introduction of epigenetic regulation of gene transcription, this review will provide an overview over recent genetic and epigenetic findings in the context of alcohol dependence focusing on human studies. Finally, we will discuss the current limitations of epigenetic studies as well as the implications of genetic and epigenetic findings for the development of better treatment and prevention strategies.

## Introduction

Alcohol dependence is a severe disorder which contributes substantially to the global burden of disease. According to the World Health Organization (WHO) 2 billion individuals worldwide consume or abuse alcohol. Alcohol consumption accounts for 3.2% of all deaths worldwide, as well as for 4% of Disability Adjusted Life Years (DALYs) which are expressed as the number of years lost due to ill-health, disability, or early death (WHO, 2002). Despite the deleterious effects of chronic alcohol consumption, efficacy of preventive and therapeutic strategies still needs to be optimized.

The heritability of alcohol dependence is well recognized and heritability estimates range between 40 and 70% [1–4]. However, the effects of the genetic risk variants for psychiatric disorders, including alcohol dependence, identified so far are small, and even after consideration of polygenetic contributions they explain only a small fraction of the estimated heritability of psychiatric disorders. One possible explanation for this “missing heritability” [5] might be the interaction between genes and environment mediated by epigenetic mechanisms.

The aim of this review is to provide an overview of recent human studies and to summarize and discuss the role and importance of genetic and epigenetic factors in alcohol dependence.

### Epigenetic mechanisms

Epigenetic mechanisms lead to functionally relevant modifications of the genome as they induce stable changes in gene expression which consequently influence the phenotypic outcome. These transcriptional control mechanisms, which can be heritable, are not accompanied by nucleotide changes in the underlying DNA sequence. Epigenetic mechanisms include histone modifications, activities of noncoding RNA, and chemical alterations of the DNA molecule itself. While the involvement of DNA methylation in transcriptional regulation is a long known phenomenon [6], the distinct role of 5-hydroxymethylation in this process still has to be investigated [7–9]. In this review, we will focus on the role of DNA methylation in epigenetic regulation of gene expression.

### DNA methylation

DNA is methylated at position 5 of the cytosine pyrimidine ring by transfer of the methyl group from S-adenosyl methionine (SAM), a reaction catalyzed by DNA methyltransferases (DNMTs). DNA methylation is a stable chemical mark which is maintained through cycles of cell division. DNA methylation mainly takes place at CpG sites, nucleotide sequences consisting of a cytosine followed by a guanine. CpG sites can be found in low frequency throughout the genome, but are enriched in so called CpG islands, which are mainly found in the promoter regions of genes near the transcription start site. Promoter DNA methylation has been strongly correlated with gene silencing, as the chemical modification of the DNA is interfering with transcription factor binding and attracts methyl-binding domain-containing proteins, such as methyl CpG binding protein 2 (MeCP2), which act as transcriptional repressors [10]. However, more recent studies suggest that the pattern of transcriptional regulation through DNA methylation is more complex than previously anticipated [11]. Traditionally it has been believed that DNA methylation patterns are important for cell fate determination and that they are fixed during embryonic development and cell differentiation and remain stable afterwards [6]. Today it is known that DNA methylation patterns are not as fixed as previously thought, but can be influenced by environmental factors and change over time [12]. Therefore, regulation of gene transcription through DNA methylation which is mediated by environmental stimuli plays a role in physiological responses as well as in adaptation of the genome to environmental requirements. Factors influencing DNA methylation are among others diet (e.g. [13]), chemicals (e.g. [14]), drugs of abuse (e.g. [15]), and even psychosocial factors like exposure to stress (e.g. [16]).

## Review

We conducted a systematic literature search using NCBI PubMed (http://www.ncbi.nlm.nih.gov/pubmed/) and keywords related to genetics and epigenetics of alcohol abuse and dependence. Genome-wide association studies in the context of alcohol dependence have been identified using the Catalog of Published Genome-wide association studies (http://www.genome.gov/gwastudies/). We included studies published prior to April 2013 in our review. The reference sections of the chosen studies were reviewed to detect additional studies we might have missed in our literature search. Genetic studies, with a strong focus on genome-wide results, will be reviewed first, followed by a section on gene-environment interactions, and finally a review of epigenetic studies in the context of alcohol dependence. This part of the review will first summarize recent candidate-gene based approaches followed by a report of the results of the two epigenome-wide studies published to date. The last section of the review will discuss recent findings on epigenetics of alcohol withdrawal.

### Genetics and epigenetics of alcohol dependence

The contribution of genetic factors to the development of alcohol dependence is high [1–4] but nevertheless, knowledge about the specific genes underlying alcohol dependence is currently very limited. The best classical candidate genes for alcohol dependence are alcohol dehydrogenase (*ADH*) and aldehyde dehydrogenase (*ALDH*) [17]. Both genes are involved in enzymatic degradation of alcohol. Recently, the first genome-wide association studies (GWAS) on alcohol dependence have been published [18–29].

These studies revealed some interesting new candidate genes for alcohol dependence including cadherin 11 (*CDH11*) [22] and cadherin 13 (*CDH13*) [22, 25], GATA binding protein 4 (*GATA4*) [25], solute carrier family 22, member 18 (*SLC22A18*) [19], and potassium large conductance calcium-activated channel, subfamily M, alpha member 1 gene (KCNMA1) [23]. Moreover, they provided further evidence for an association between *ADH* and *ALDH* and alcohol dependence [20, 23, 25].

Results of a recent study by Yan et al. [30] investigating the predictive value of the cumulative impact of multiple genetic variants identified in previous candidate as well as genome-wide studies, support a polygenic model for alcohol dependence. This polygenic model of inheritance involves many genetic variants of small effects to contribute to the risk of alcohol dependence. Similar models of inheritance have been found for schizophrenia and bipolar disorder [31] and might apply to many other common complex diseases. However, this study also showed that a positive family history of alcohol dependence was a better predictor for alcohol dependence than the sum of genetic risk variants. This finding illustrates that the genetic risk variants identified so far can only explain a small fraction of the heritability of alcohol dependence.

One possible explanation for this phenomenon of “missing heritability” [5] is that beside the genome itself, the cross-talk between the genome and the environment is implicated in the etiology of alcohol dependence. Gene-environment interactions have been reported for the serotonin transporter (*5-HTT*) [32–35], the dopamine receptor (*DRD2*)/ankyrin repeat and kinase domain containing 1 (*ANKK1*) [36–38], catechol-O-methyltransferase (*COMT*) [39, 40], monoamine oxidase (*MAOA*) [41–43], GABA_A_ receptor (*GABRA2*) [44], corticotrophin releasing hormone receptor 1 (*CRHR1*) [45–48].

Gene-environment interactions can be mediated by epigenetic mechanisms and evidence is emerging that alcohol consumption is one environmental factor which may alter epigenetic signatures and thus related gene expression levels. Altered gene expression in brain reward regions after alcohol intake has been reported, which suggests that individual genes are differentially regulated following alcohol consumption [49]. In actively drinking individuals, or individuals in early stages of alcohol abstinence, increased levels of homocysteine have been found [50–54]. Homocysteine plays an important role in DNA methylation as it is metabolized to methionine, which is then activated to S-adenosyl methionine (SAM) by ATP [55, 56]. SAM is the most important methyl group donor in transmethylation reactions in vertebrates, including DNA methylation. Consequently, elevated homocysteine levels have been found to be associated with increased levels of global as well as gene-specific DNA methylation in alcohol dependent patients [57, 58]. In contradiction to this finding in alcohol dependent patients, other studies investigating human subjects reported DNA hypomethylation to be associated with hyperhomocysteinemic states [59–63].

Alterations in promoter DNA methylation levels in alcohol dependent patients have been reported for homocysteine-induced ER protein (*HERP*), alpha synuclein (*SNCA*), vasopressin (*AVP*), nerve growth factor (*NGF*), atrial natriuretic peptide (*ANP*), N-methyl-D-aspartate 2b receptor subtype (*NR2B*), *MAOA*, *5-HTT*, μ-opioid receptor (*OPRM1)*, prodynorphin (*PDYN*), pro-opiomelanocortin (*POMC*), dopamine transporter (*DAT*) [64–75].

While some of these studies reported hypermethylation in alcohol dependent patients (e.g. [64, 66, 67, 70, 71]), other reported a hypomethylation of the gene region investigated (e.g. [65, 71]).

Recently, Zhang et al., [76] published an array-based study investigating methylation levels of 384 CpGs in 82 candidate genes. The candidate genes have been selected based on their involvement in brain neurotransmission systems (dopaminergic, opiodergic, serotonergic, GABAergic/glutamatergic, cholinergic and cannabinoidergic), in alcohol metabolism, DNA methylation, or in signal transduction for alcohol reward and reinforcement, or they have been found associated with alcohol dependence in previous genetic studies. CpGs in gamma-aminobutyric acid receptor subunit beta-3 (*GABRB3*), pro-opiomelanocortin (*POMC*), neural cell adhesion molecule 1 (*NCAM1*), dopamine receptor D4 (*DRD4*), methyl-CpG-binding domain protein 3 (*MBD3*), 5-hydroxytryptamine receptor 3A (*HTR3A*) and 2B (*HTR2B*), and glutamate (NMDA) receptor subunit zeta-1 (*GRIN1*) were found to be differentially methylated in alcohol dependent patients compared to control subjects, but only the result for one CpG site in the promoter region of *HTR3A* withstood correction for multiple testing.

The same CpG site showed nonsignificantly higher methylation levels in a small replication sample, where only 6 CpGs in *HTR3A* have been investigated. To confirm this preliminary finding of differential methylation in *HTR3A* in alcohol dependence, additional studies will be necessary.

While these studies concentrated on candidate genes, the first hypothesis free genome-wide investigations of the effect of alcohol on DNA methylation have been currently published:

Philibert et al. conducted a genome-wide study on the effect of recent alcohol use on the epigenome [77]. Here, the authors compared DNA methylation of females which did not consume alcohol in the last 6 months to methylation patterns of females consuming alcohol mildly (use of alcohol in between 1 and 8 weeks in the last 6 month), moderately (use of alcohol in between 9 and 25 weeks in the last 6 month), or heavily (alcohol use in every week in the last 6 month). By doing this, they found the overall degree of methylation to be (nonsignificantly) positively associated with the amount of alcohol consumed. When the moderate and heavy drinkers are pooled together and compared to the non-consuming participants, a probe located in the active BCR-related (*ABR*) gene, and in the 5` untranslated region of the Bladder cancer-associated protein gene (*BLCAP*) reached genome-wide significance after correction for multiple testing. However, only 4 of the heavy drinkers investigated met the criteria for a lifetime diagnosis of alcohol dependence, therefore this result needs further replication in a sample of alcohol dependent patients compared to control individuals.

The only epigenome-wide study investigating alcohol dependent subjects has been published by Zhang et al., early this year [78]. In contrast to Phillibert et al., Zhang et al., found the global methylation level, as well as the majority of significantly differentially methylated CpG sites, including the 50 most significant ones, to be hyopmethylated in patients compared to healthy controls. However, neither of these studies included a measurement of homocysteine levels, which would have been necessary to answer the question, whether DNA methylation on an epigenome-wide level is associated with the assumed elevated homocysteine levels in alcohol dependent patients.

Further studies addressing this question in a hypothesis-free genome-wide approach have to follow to clarify this issue as well as to elucidate the effects of alcohol dependence on the epigenome.

In addition to the elevated homocysteine levels in actively drinking individuals [50–54], during alcohol withdrawal, decreasing homocysteine levels have been observed [50, 51, 53, 79]. Under the assumption that elevated homocysteine levels are associated with increased levels of DNA methylation in alcohol dependent patients [57, 58], a plausible hypothesis is that methylation levels also decrease during alcohol withdrawal. The first studies investigating the effects of alcohol withdrawal on DNA methylation have been conducted applying a candidate gene approach [65, 68, 69]. These studies reported conflicting results regarding the hypothesis of decreasing DNA methylation levels during alcohol withdrawal. Biermann et al., [65] found a decrease of *NR2B* promoter methylation during withdrawal, although this decrease did not reach significance. In contrast to that, Heberlein et al., [68] investigating *NGF*, reported an increase in promoter methylation, and in our own study, we [69] did not detect an effect of alcohol withdrawal on *DAT* promoter methylation.

## Conclusions

There is a growing body of evidence highlighting the importance of genetic and epigenetic factors in the development and maintenance of alcohol dependence. Nonetheless, by investigating epigenetic factors in alcohol dependence, some limitations have to be kept in mind.

Most of the studies conducted thus far used peripheral tissue instead of brain samples, mostly because access to high quality brain material of well characterized alcohol dependent subjects is very limited. While this is not an issue in genetic studies, it might be a limitation in epigenetic research as epigenetic regulation is tissue- and cell-type specific. However, as alcohol acts not only on the brain but on the whole body, it could by hypothesized that there might be a significant overlap between methylation changes in the brain and blood, despite the cell-type specific nature of DNA methylation patterns. Moreover, the use of peripheral tissue is a valid approach as it is necessary for the identification of biomarkers with potential clinical usage. To identify those biomarkers, we need a material which is easily accessibly and can be studied in large samples of patients and control individuals. Peripheral blood is an adequate and convenient tissue for that purpose.

As the field of epigenetic research is relatively new, laboratory technologies still need to be optimized to provide an unbiased method which I) requires small amounts of input samples, II) is cost effective, and III) will be able to reliably detect epigenetic marks with single nucleotide resolution. In parallel with the improvement of laboratory techniques, new analytical methods and guidelines for quality control and analysis need to be developed. The novelty of epigenetic research in alcohol dependence also explains the relatively small number of studies published so far. In addition, a limited number of research groups currently conduct epigenetic studies in the field of alcohol dependence. The limitation of the data available could explain the still heterogeneous nature of the results regarding epigenetic effects of alcohol dependence and withdrawal. Furthermore, the literature is likely biased for positive associations, as negative findings are infrequently published. Therefore, further genetic and epigenetic research investigating large, homogenous groups of patients and controls which are matched as carefully as possible for confounding factors such as age, sex, smoking and other environmental factors potentially influencing epigenetic regulation are necessary. Despite the previously mentioned difficulties that need to be addressed in the near future, epigenetic studies have a great potential to contribute to a better understanding of the biological mechanisms underlying alcohol dependence. A deeper insight into the epigenetic regulation of alcohol dependence, withdrawal and relapse is needed to develop better treatment and prevention strategies. In addition, a better understanding of epigenetic regulation might help to detect individuals at a higher risk to develop alcohol dependence and/or identify alcohol dependent patients with a higher risk of relapse.

However, it has to be kept in mind that studies investigating epigenetics of withdrawal are currently very limited so conclusions should therefore be drawn with caution.
